# Effects of paternal obesity on maternal-neonatal outcomes and long-term prognosis in adolescents

**DOI:** 10.3389/fendo.2023.1114250

**Published:** 2023-03-30

**Authors:** Yingying Lin, Zhiwei Chen, Qinfang Qian, Yanxia Wang, Xiaoyan Xiu, Ping Ou, Jiaoning Fang, Guobo Li

**Affiliations:** ^1^ Department of Healthcare, Fujian Maternity and Child Health Hospital, College of Clinical Medicine for Obstetrics & Gynecology and Pediatrics, Fujian Medical University, Fuzhou, China; ^2^ Department of Obstetrics and Gynecology, Fujian Maternity and Child Health Hospital, College of Clinical Medicine for Obstetrics & Gynecology and Pediatrics, Fujian Medical University, Fuzhou, China; ^3^ Department of Child Healthcare Centre, Fujian Maternity and Child Health Hospital, College of Clinical Medicine for Obstetrics & Gynecology and Pediatrics, Fujian Medical University, Fuzhou, China

**Keywords:** paternal obesity, BMI, maternal-neonatal outcomes, long-term, prognosis

## Abstract

**Objective:**

This study evaluated whether paternal body mass index (BMI) before pregnancy was a risk factor for maternal-neonatal outcomes and long-term prognosis in offspring.

**Methods:**

This study included 29,518 participants from eight cities in Fujian, China using a stratified cluster random sampling method from May to September 2019. They were divided into four groups based on paternal BMI. Univariate and multivariate logistic regression were used to explore the relationship between paternal BMI groups, maternal-neonatal outcomes, and long-term prognosis in offspring. Further subgroup analysis was conducted to examine the stability of the risk.

**Results:**

The incidences of hypertensive disorder complicating pregnancy (HDCP), cesarean delivery, gestational weight gain (GWG) over guideline, and macrosomia were significantly higher in the paternal overweight and obesity group. Importantly, this study demonstrated that the incidence of asthma, hand-foot-and-mouth disease (HFMD), anemia, dental caries, and obesity of adolescents in paternal obesity increased. Furthermore, logistic regression and subgroup analysis confirm paternal obesity is a risk factor for HDCP, cesarean delivery, and macrosomia. It caused poor long-term prognosis in adolescents, including asthma, dental caries, and HFMD.

**Conclusions:**

Paternal obesity is a risk factor for adverse maternal-neonatal outcomes and poor long-term prognosis in adolescents. In addition to focusing on maternal weight, expectant fathers should pay more attention to weight management since BMI is a modifiable risk factor. Preventing paternal obesity can lead to better maternal and child outcomes. It would provide new opportunities for chronic diseases.

## Introduction

Globally, more than 700 million people were diagnosed as overweight or obese in 2015, which continuously increased in most other countries (1). With a rapidly growing trend of obesity incidence, the proportion of obese men has risen more than three times (2). It is estimated that the number of overweight and obesity will reach 1.3 billion and 573 million worldwide by 2030, of which 43% and 21% will live in Asia (3). Especially in developed countries, nearly 60% of adult men are overweight or obese (4). Being overweight and obese are the fifth leading risk of death globally and are major risk factors for many diseases, including diabetes, cardiovascular disease, and cancer. It is reported that nearly 2.8 million deaths annually are related to obesity. In addition, 44. 0% of diabetes, 23. 0% of specific cancers are related to overweight and obesity (5–7). Overweight and obesity have been considered a threat to public health worldwide, bringing a vast economic and social burden (8, 9). Recent studies have shown that obesity affects patients’ health and harms future generations in the short and long term (10, 11).

Parental obesity can cause damage to the growth and development, metabolism, and nervous system of the fetus and long-term outcomes of offspring after birth, which seriously affects the healthy growth of the progeny. It is also a high-risk factor for metabolic diseases in adulthood, leading to a vicious circle of obesity and other metabolic disorders in the population. The impact of maternal obesity on pregnancy outcomes and newborn health has been widely studied. Numerous studies have pointed to maternal obesity leading to an increased incidence of pregnancy complications, including gestational diabetes (GDM) (12), preeclampsia (13), hypertension, postpartum depression (14), cesarean section, preterm birth, and poor neonatal outcomes including perinatal death, macrosomia, and fetal defects (15, 16). However, the impact of paternal obesity on pregnancy and child health remains limited.

Obesity is the basis of metabolic diseases regulated by genetic factors and the acquired environment, and the two can also interact (17, 18). Recently, studies have suggested that male obesity affects their health and has adverse effects on offspring through sperm cells (19). Animal model of diet-induced obese rats suggests that paternal obesity can lead to impaired islet function in offspring, resulting in insulin homeostasis imbalance (20, 21). Chowdhury.SS et al. (22) suggest that paternal obesity can lead to early renal impairment in offspring. In addition, Terashima M et al. (23)further indicated that paternal obesity induced by a high-fat diet also has a particular passage effect on the liver genes of male offspring. They found that paternal obesity could cause fat deposition in the liver of their male offspring mice, affecting the expression of genes such as Mt1 and Mt2. Currently, cohort studies on the impact of paternal obesity on the health of offspring have also been reported. Chen et al. (24) found that paternal body mass index (BMI) was positively correlated with birth weight, double top diameter, chest circumference, abdominal circumference, and cord blood cortisol in their male newborns but had no effect on female offspring. However, Figueroa-Colon R et al. (25) pointed out that the father’s total amount and percentage of body fat are the main predictors of changes in the total amount and percentage of body fat before menstruation in female offspring. Unfortunately, there are few clinical studies on the effects of paternal obesity on offspring health, and the differences in the impact of paternal obesity on offspring of different sexes are highly controversial.

In the present study, we analyze the associations between paternal obesity and the clinical outcomes with maternal-neonatal outcomes and long-term prognosis with children using a large historical cohort study. We aimed to evaluate whether paternal obesity is a valuable risk factor influencing pregnancy complications and short-term and long-term neonatal outcomes.

## Material and methods

### Participants

**Figure 1:**
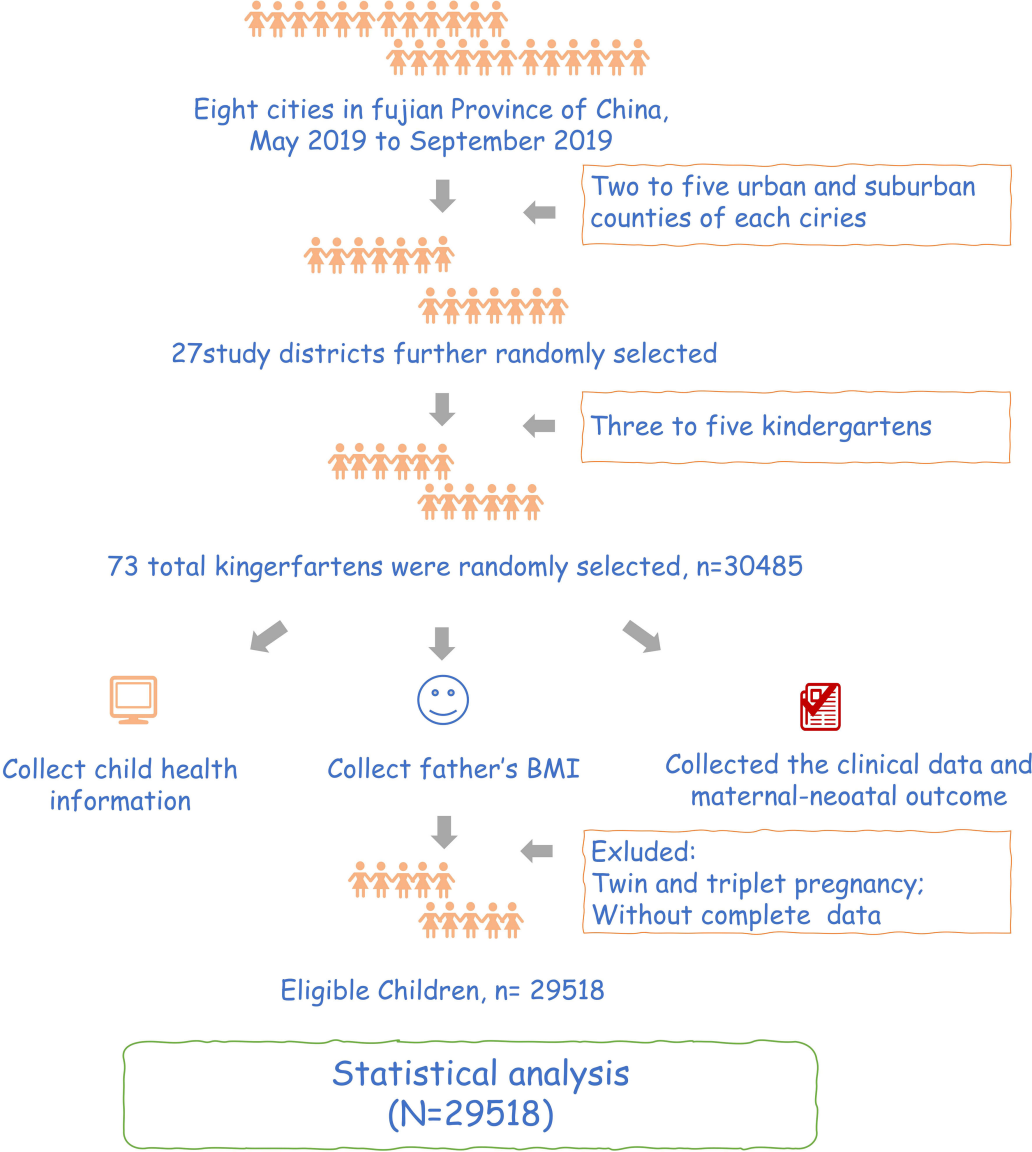
Flowchart of participants in the study.

This study is a historical cohort study. We conducted this study in Fujian, China, from May to September 2019 using stratified cluster random sampling. We selected the cities from 8 cities in Fujian province and randomly selected 1 to 4 districts and counties from each chosen city. Finally, in the survey of each section and county, with kindergarten as a unit, from the cluster randomly selected 3 to 5 kindergartens. We collected the father’s BMI, clinical data, maternal-neonatal outcome, and early childhood growth and development information in preschool children. Participants should meet the following criteria:1) singleton pregnancy; 2) have complete data; 3)signed informed consent forms. After excluding participants with twin and triplet pregnancies and without exclusive data, we finally included 29,518 participants in this study (**Figure 1**). The study was conducted following the Declaration of Helsinki and approved by the Institutional Review Board of Fujian Maternity and Child Health Hospital (protocol code:117. April 3, 2019). All individuals participating in this study signed written informed consent.

### Clinic data collection

All participants’ detailed clinical data were collected from computerized obstetric records, neonatal databases, and handwritten records. The basic information contains demographic characteristics and maternal-neonatal outcomes. Demographic factors include maternal age, paternal age, gravity, parity, maternal BMI, and paternal BMI. The father’s weight within six months before pregnancy is obtained based on the current year’s medical examination record or inquiry. The child’s illness is obtained through kindergarten health and electronic medical records. All information collection and recording were recorded by uniformly trained maternal and child health professionals. In addition, maternal-neonatal outcomes were recorded after the delivery, including pregnancy complications, maternal weight gain during pregnancy, and gestational age at delivery.

### Definition

This study adopted the BMI in the guidelines for preventing and controlling overweight and obesity in Chinese adults. The BMI = weight (kg)/height^2^ (m^2^). According to the suggestions (26), BMI is divided into four groups: BMI < 18.5 is underweight, 18.5 ≤ BMI < 24 is average, 24 ≤ BMI < 28 is overweight, and BMI ≥ 28 is obesity. “Weight value for height” was used to evaluate children’s physical development level (27). Small for gestational age (SGA) refers to the birth weight being less than the lightest 10% of the birth weight of all babies born within the same gestation week. Macrosomia indicates the birth weight of a newborn equal to or greater than 4000 grams. Preterm birth means birth before 37 weeks of pregnancy. Gestational diabetes mellitus (GDM) is any glucose intolerance with onset or first recognition during pregnancy. Hypertensive disorder complicating pregnancy (HDCP) means previously normotensive women should have a systemic systolic blood pressure≥140 mmHg and a diastolic blood pressure ≥90 mmHg after 20 weeks of gestation.

### Statistical analysis

SPSS version 26.0 (IBM, Armonk, NY, USA) and R software version 4.2.2 (R Development Core Team, Oct 2022; http://www.r-project.org) was used for all statistical analyses. The qualitative data were presented as rates and used to analyze the differences in overweight and obese rates of different groups with the chi-square test. The cunting data were expressed as means ± standard deviation and diagnosed with t-tests. Univariate logistic regression was used to compare pregnancy complications, maternal-neonatal outcomes, and long-term neonatal outcomes in different paternal BMI groups. Multivariate regression analysis was used to adjust for confounding variables known to independently affects. The odds ratio (OR) and 95% confidence interval (CI) were calculated to identify risk factors and assess their impact. A P-value less than 0.05 was considered statistically significant.

## Results

### Basic information about the population

**Figure 2:**
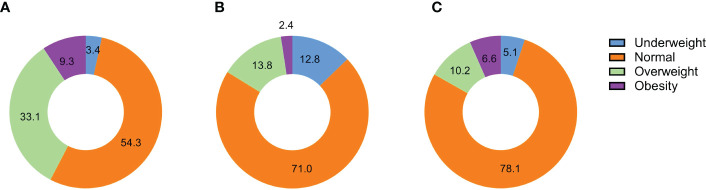
The distribution of BMI groups for different types of participants. **(A)**. The distribution of BMI groups in fathers; **(B)**. The distribution of BMI groups in mothers; **(C)**. The distribution of BMI groups in children;.

Finally, 29,518 participants were included in this study. The progress of the study.was shown in **Figure 1**. Grouping according to the paternal BMI, a total of 996 (3.4%) were in the underweight group, 16,028 (54.3%) were in the normal BMI group, 9,761 (33.1%) were in the overweight group, and 2,733 (9.3%) were in the obesity group. Moreover, maternal BMI was also divided into four groups, including 3,782 in underweight (12.8%), 20,955 in normal (71.0%), 4,071 in overweight (13.8%), and 710 in obesity (2.4%). In addition, most children were classified as normal (78.1%), followed by overweight (10.2%), obese (6.6%), and underweight (5.1%) based on weight value for height (**Figure 2**).

The individuals’ essential characteristics are presented in **Table 1**, stratified into four groups based on paternal BMI. The mean paternal age was 35.1 ± 4.9 years, and the mean maternal age was 33.0 ± 4.4 years. The paternal age, paternal education, maternal age, maternal BMI, and annual family income were significantly related to different paternal BMI groups.

**Table 1:** Clinical characteristics of the study participants^a^.

Variables	Paternal BMI (kg/m^2^)				*P* value^b^
	Underweight (n = 996)	Normal (n = 16028)	Overweight (n = 9761)	Obesity (n = 2733)	
Paternal age (y)	33.11 ± 4.49	35.00± 4.93	35.44± 4.94	34.81 ± 4.82	<0.001
Paternal education (%)					<0.001
High school and below	498 (50.00)	8149 (50.84)	5355 (54.86)	1592 (58.25)
Bachelor’s degree	438 (43.98)	6998 (43.66)	3968 (40.65)	1050 (38.42)
Master degree or above	60 (6.02)	881 (5.50)	438 (4.49)	91 (3.33)
Maternal age (y)	33.37 ± 4.27	33.17 ± 4.37	32.89 ± 4.34	32.24 ± 4.40	<0.001
Maternal education (%)					0.073
High school and below	530 (53.21)	8456 (52.76)	5109 (52.34)	1390 (50.86)
Bachelor’s degree	415 (41.67)	6942 (43.31)	4301 (44.06)	1241 (45.41)
Master degree or above	51 (5.12)	630 (3.93)	351 (3.60)	102 (3.73)
Gravity (%)					0.400
<2	520 (52.21)	8804(54.93)	5368 (55.00)	1503 (54.99)
≥2	476 (47.79)	7224 (45.07)	4393 (45.00)	1230(45.01)
Parity (%)					0.142
<2	435 (43.67)	7592 (47.37)	4631 (47.44)	1300 (47.57)
≥2	561 (56.33)	8436 (52.63)	5130 (52.56)	1433 (52.43)
Maternal BMI					<0.001
Underweight	120 (12.05)	1973(12.31)	1302 (13.34)	387 (14.16)
Normal	692 (69.48)	11343 (70.77)	6995 (71.66)	1925(70.44)
Overweight	158 (15.86)	2317(14.46)	1258 (12.89)	338 (12.36)
Obesity	26 (2.61)	395 (2.46)	206 (2.11)	83(3.04)
Annual family income ¥, [n(%)]					0.022
≤3,000	76 (7.63)	1176 (7.33)	665 (6.81)	202 (7.39)
3,000-8,000	353 (35.44)	6076 (37.91)	3644 (37.33)	951 (34.80)
*>*8,000	567 (56.93)	8776 (54.75)	5452 (55.85)	1580 (57.81)

BMI, body mass index;

a Values are mean ± SD for continuous variables or n (%) for categorical variables.

b P values were derived from one-way ANOVA for continuous variables and chi-square test for categorical variables.

### Effects of paternal BMI on maternal-neonatal outcomes

The results of maternal and neonatal outcomes in different paternal BMI groups are shown in **Table 2**. It is reported that the incidences of cesarean delivery, gestational weight gain (GWG) over guidelines, and macrosomia in the paternal obesity group and the overweight group were significantly higher than in the normal BMI group. Also, the incidences of HDCP in the paternal obesity group were significantly higher than in the normal BMI group.

**Table 2:** Comparison of maternal-neonatal outcomes in different paternal BMI groups.

Variables		Underweight	Normal	Overweight	Obesity	*P* value^b^
GDM	n (%)	50 (5.00)	906 (5.70)	539 (5.50)	159 (5.80)	0.779
	P^a^	0.400	Reference	0.658	0.730	
HDCP	n (%)	20 (2.00)	272 (1.70)	195 (2.00)	99 (3.60)	<0.001*
	P^a^	0.464	Reference	0.079	<0.001*	
Cesarean delivery	n (%)	397 (39.90)	5849 (36.50)	3713 (38.00)	1085 (39.70)	<0.001*
	P^a^	0.033*	Reference	0.013*	0.001*	
GWG over guidelines	n (%)	79 (7.90)	1116 (7.00)	758 (8.00)	247 (9.00)	0.001*
	P^a^	0.064	Reference	0.001*	0.002*	
Premature birth	n (%)	102 (10.20)	1566 (9.80)	905 (9.3)	266(9.70)	0.519
	P^a^	0.628	Reference	0.187	0.951	
SGA	n (%)	100 (10.00)	879 (5.50)	554 (5.70)	171 (6.30)	<0.001*
	P^a^	<0.001*	Reference	0.515	0.105	
Macrosomia	n (%)	79 (7.90)	1108 (6.90)	742 (7.60)	245 (9.00)	0.001*
	P^a^	0.221	Reference	0.038*	<0.001*	

BMI, body mass index; GDM, gestional diabetes mellitude; HDCP, hypertensive disorder complicating pregnancy; GWG, gestational weight gain; SGA, small for gestational age;

a The p-values taken from univariate logistic regression compared with the referent (normal BMI).

b P values for trend across categories of different paternal BMI.

*P<0.05 was considered statistically significant.

In addition, the incidences of cesarean delivery and SGA rate in the underweight paternal group were significantly higher than in the normal BMI group.

### Effects of paternal BMI on long-term neonatal outcomes

**Table 3** compares long-term neonatal outcomes in different paternal BMI groups. The results showed that the incidence of asthma, hand-foot-and-mouth disease (HFMD), anemia, dental caries, and obesity of adolescents in the paternal obesity group showed an increasing trend compared with the normal paternal BMI group. And then, the incidence of asthma, HFMD, and obesity of adolescents in the overweight paternal group was significantly higher than in the normal BMI group but not in the underweight group. Contrarily, the incidence of anemia in the underweight but not the overweight group was significantly higher than in the normal BMI group.

**Table 3:** Comparison of long-term neonatal outcomes in different paternal BMI groups.

Variables		Underweight	Normal	Overweight	Obesity	*P* value^b^
Asthma	n (%)	17(1.70)	257 (1.60)	201 (2.10)	73 (2.70)	0.001*
	P^a^	0.801	Reference	0.007*	<0.001*	
Allergic diseases	n (%)	212 (21.30)	3367 (21.00)	2013 (20.6)	572 (20.90)	0.885
	P^a^	0.834	Reference	0.462	0.927	
HFMD	n (%)	71 (7.10)	968 (6.00)	666 (6.80)	198(7.20)	0.016*
	P^a^	0.164	Reference	0.012*	0.016*	
Anaemia	n (%)	77 (7.70)	603 (3.80)	393 (4.00)	125(4.60)	<0.001*
	P^a^	<0.001*	Reference	0.286	0.043*	
Dental caries	n (%)	154 (15.50)	2141 (13.40)	1351 (13.80)	421 (15.40)	0.014*
	P^a^	0.060	Reference	0.272	0.004*	
Visual impairment	n (%)	44 (4.40)	726 (4.50)	400 (4.100)	128 (4.70)	0.346
	P^a^	0.869	Reference	0.100	0.721	
Obesity in Adolescents	n (%)	71 (7.10)	979 (6.10)	673 (6.90)	216 (7.90)	0.017*
	P^a^	0.425	Reference	0.024*	0.007*	

BMI, body mass index; HFMD, hand-foot-and-mouth disease;

a The p-values taken from univariate logistic regression compared with the referent (normal BMI).

b P values for trend across categories of different paternal BMI.

*P<0.05 was considered statistically significant.

### Paternal obesity is a risk factor in predicting poor maternal-neonatal outcomes and poor long-term prognosis in adolescents

The results of the multivariate logistic analysis demonstrated that paternal obesity would significantly increase the incidence of HDCP, a cesarean delivery, and GWG over guidelines and macrosomia of participants. Moreover, it also caused some poor long-term prognoses in children, including asthma, HFMD, dental caries, and obesity. Furthermore, participants in the overweight paternal group had a higher rate of cesarean delivery and GWG over guidelines. Notably, the children had a higher incidence of asthma, HFMD, and obesity. Also, we found that the underweight paternal group is a risk factor for the incidence of SGA and anemia (**Table 4**).

**Table 4:** Odds ratio for the associations between paternal BMI and pregnancy outcomes and long-term neonatal outcomes.

Variables	Odds ratio (95% CI)			
	Underweight	Normal	Overweight	Obesity
HDCP	1.16 (0.73-1.84)	[Reference]	1.17 (0.97-1.41)	2.13 (1.69-2.70)*
Cesarean delivery	1.13 (0.99-1.29)	[Reference]	1.06(1.01-1.12)*	1.13 (1.04-1.23)*
GWG over guidelines	1.12 (0.99-1.24)	[Reference]	1.07 (1.02-1.11)*	1.10 (1.03-1.17)*
SGA	1.97 (1.58-2.46)*	[Reference]	1.05(0.94-1.18)	1.18 (0.99-1.40)
Macrosomia	1.20 (0.94-1.53)	[Reference]	1.09 (0.99-1.21)	1.30 (1.12-1.50)*
Asthma	0.95 (0.58-1.57)	[Reference]	1.26 (1.04-1.51)*	1.57 (1.21-2.05)*
HFMD	1.11 (0.87-1.43)	[Reference]	1.13 (1.02-1.25)*	1.17 (1.01-1.38)*
Anaemia	2.08 (1.62-2.67)*	[Reference]	1.05 (0.92-1.19)	1.16 (0.95-1.41)
Dental caries	1.13(0.95-1.36)	[Reference]	1.04 (0.96-1.12)	1.17 (1.04-1.31)*
Obesity in Adolescents	1.04 (0.94-1.16)	[Reference]	1.05 (1.01-1.09)*	1.09 (1.03-1.17)*

BMI, body mass index; HDCP, hypertensive disorder complicating pregnancy; GWG, gestational weight gain; SGA, small for gestational age; HFMD, hand-foot-and-mouth disease.

*P < 0.05 was considered statistically significant.

Maternal BMI performed subgroup analysis. The test for interaction showed that the effect of paternal obesity on cesarean section was significantly affected by maternal obesity(*P*<0.05), but there was no statistical significance on SGA, Macrosomia, HDCP, anemia, dental caries, asthma, and HFMD (*P*>0.05).

**Figure 3:**
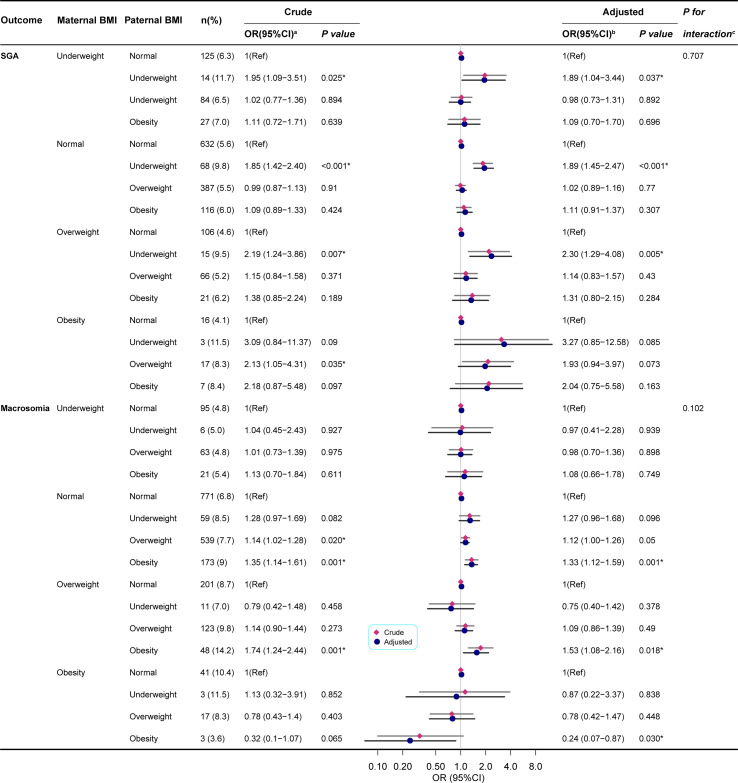
Paternal BMI and the risk of SGA/Macrosomia was analyzed by logistic regression, stratified by maternal BMI. BMI, body mass index; SGA, small for gestational age; OR, odds ratio;95% CI, 95% confidence interval of the estimated trend. (a) Univariate logistic regression compared with the referent (norma paternal BMI). (b) Estimated using multivariate logistic regression. Model for SGA/Macrosomia adjusted for child sex, child age, paternal age, paternal education, maternal age, maternal education, parity, gravidity, annual family income, HDCP, cesarean delivery, GWG. And macrosomia is also adjusted for gestational age. (c) *P* values for interaction showed the interaction effect of paternal obesity and maternal obesity on the risk of SGA/Macrosomia. *P < 0.05 was considered statistically significant.

For maternal BMI underweight, normal, and overweight, paternal underweight significantly increased the incidence of SGA, while for maternal obesity, paternal BMI was not statistically significant for SGA. For maternal BMI normal and overweight, paternal obesity significantly increased the incidence of Macrosomia (**Figure 3**).

For maternal BMI underweight, normal, and obese, paternal obesity significantly increased the risk of HDCP, but there was no statistical significance in paternal BMI for overweight mothers. For maternal BMI overweight and obesity, paternal obesity significantly increased the risk of cesarean delivery (**Supplementary Table 1**).

For maternal BMI underweight, normal, and overweight, paternal underweight significantly increased the risk of anemia. In contrast, paternal obesity significantly increased the risk of anemia and dental caries in the normal maternal group. For maternal BMI underweight, paternal underweight significantly increased the risk of dental caries. The group of overweight or obese parents can increase the risk of anemia (**Supplementary Table 2**).

For maternal BMI underweight, paternal obesity significantly increased the risk of asthma. For maternal BMI normal, paternal overweight or obesity significantly increased the risk of HFMD (**Supplementary Table 3**).

## Discussion

In this study, we found that paternal obesity before pregnancy was significantly related to an increased risk of macrosomia in babies and pregnancy complications in mothers, including HDCP, cesarean delivery, and GWG over guidelines. It is similar to the results of Lin J et al. (28). Moreover, the incidences of SGA in offspring were significantly higher in the underweight paternal groups. Therefore, this study suggested that paternal obesity before pregnancy may be a risk factor for macrosomia in offspring and pregnancy complications, including HDCP, cesarean delivery, and GWG over guidelines in the mother. And paternal BMI underweight before pregnancy may be a risk factor for SGA in offspring. It is consistent with research by Li J et al. (29). Importantly, this study has pointed out a significant problem. A close relationship between paternal BMI level and long-term neonatal outcomes in adolescents may exist. It demonstrated an increasing trend in the incidence of asthma, HFMD, anemia, dental caries, and obesity of adolescents in the paternal obesity group. Johnson W et al. also suggest paternal obesity is related to childhood obesity (30).

Professor David Barker proposed the “Barker hypothesis” based on discovering that maternal malnutrition before pregnancy is associated with permanent glucose-insulin homeostasis disruption in offspring (31). This hypothesis was later renamed “Developmental, Origins of Health, and Disease (DOHaD).” It refers to a child’s health and disease status later in growth or adulthood, significantly related to its environment before, during, or after pregnancy. It mainly emphasizes the role of noncommunicable diseases (NCDs), including obesity, type II diabetes, and cardiovascular disease. However, much research has also focused on cancer, mental health, and immune dysfunction. As a hot research field in the current medical area, DoHaD theory emphasizes the intrauterine environment’s influence during pregnancy on the fetus’s long-term and multi-generational health. It has important guiding significance for the pregnancy health of expectant mothers. Many articles on the impact of maternal exposure on the health of offspring have led to an important role in maternal health education and advocacy. However, the incidence of these diseases remains high, suggesting that other factors play an important role in addition to the influence of maternal factors on offspring during pregnancy. Research indicates paternal obesity may negatively affect fetal development (32, 33). Evidence shows paternal obesity also predisposes offspring to more susceptibility to metabolic diseases later in life (34, 35).

Some studies emphasize the necessity to explore and recognize fathers’ contribution to their descendants’ health in the hypothesis of the developmental origin of health and disease (36). Unlike DOHaD, they defined this new concept as the Paternal Origins of the Health and Disease paradigm (POHaD) (37). Studies have shown that paternal obesity can cause damage to the offspring’s representation through epigenetic modification of sperm. Animal experiments have shown that alterations in sperm DNA methylation can be maintained after fertilization and throughout the early embryo and that the defective gene can be passed on to offspring (38). Other studies have pointed out that the high-fat diet of parent mice will affect the transmission of small RNA fragments (tsRNA) to offspring, affecting the expression of 28 metabolism-related genes such as Gm8773 and Alx4 (39). Similarly, studies have shown dysfunction can lead to various inherited imprinting disorders. SATO et al. (40) For example, the misprinted gene in male sperm can pass the defective gene to offspring through DNA methylation. In addition, epidemiological research has shown that the methylation rate of sperm in some imprinted gene loci in obese men is significantly reduced. The children of obese parents have methylation changes in the part of the imprinted region (41, 42). Compared to maternal programming, the latest studies demonstrated the mechanisms of epigenetic inheritance of obesity-evoked metabolic and neurobiological changes through the paternal germline. It might also substantially affect their offspring (43, 44). Dupont C et al. noted that the concept of paternal transmission [Paternal Origins of Health and Diseases (POHaD)] has emerged, stressing the impact of paternal overweight or obesity on offspring’s health and development (45). The effect of paternal obesity on the health status of children has been preliminarily established, but the mechanism research is still in its infancy. Therefore, the development of epigenetics lays the foundation for studying the mechanism of paternal obesity on offspring and provides a new model for cross-generational genetic treatment of obesity and related diseases.

This study also showed by logistic regression analysis that paternal obesity would significantly increase the incidence of HDCP, a cesarean delivery, GWG over guidelines, and macrosomia. Notably, one interesting finding of this study is that paternal obesity also caused poor long-term prognoses in adolescents, including asthma, HFMD, dental caries, and obesity. Furthermore, participants in the overweight paternal group had a higher rate of cesarean delivery, GWG over guidelines, asthma, HFMD, and obesity. We found that the underweight paternal group is a risk factor for the incidence of SGA. Moreover, anemia in adolescents significantly increased. These results suggest that paternal obesity increases the frequency of extreme adverse events.

Further subgroup analysis was conducted to explore the stability of the risk. After controlling for several maternal and paternal factors, low paternal weight was found to be a risk factor for SGA and childhood anemia. In contrast, paternal obesity was associated with an increased risk of Macrosomia, HDCP, cesarean delivery, childhood dental caries, asthma, and HFMD. The results of the subgroup analysis showed that the direction of the risk association was consistent, indicating good stability. The interaction test showed that the influence of paternal obesity on cesarean section was significantly influenced by maternal obesity.

There was a correlation between paternal BMI before pregnancy and maternal and child outcomes and long-term health. The risk of maternal and child poor outcomes from paternal obesity, remained statistically significant even after controlling for other traditional risk factors for both fathers and mothers. According to the study results of Yang et al., paternal overweight and obese before pregnancy were significantly associated with the risk of macrosomia compared with the normal paternal weight, which was consistent with our results (46). Chen et al. (24) found that paternal body mass index (BMI) was positively correlated with birth weight, double top diameter, chest circumference, abdominal circumference, and cord blood cortisol in their male newborns but had no effect on female offspring by animal tests. A growing number of animal studies suggest that fathers also play a vital biological role in fetal programming, but until now, only a few human studies have supported the concept. Therefore, cohort studies are needed to confirm whether the findings are similar in humans.

Mothers are the most critical population during pregnancy and childbirth, but we ignore the vital role paternal factors can play in improving pregnancy outcomes and offspring health. To date, no country has issued separate guidelines for managing paternal pregnancy. Therefore, more cohort studies with large samples are needed to explore the impact of paternal factors during pregnancy on maternal-neonatal outcomes and offspring health. This study included a large sample size from eight cities in Fujian Province, which is well represented. In addition to collecting information on maternal and newborn pregnancy outcomes, we assessed the long-term health of these offspring in preschool age, which had not been seen in previous studies. However, the study also has several limitations. Firstly, we obtained data from self-reporting paternal height and weight, increasing the bias risk. But the weight of adults does not change much in 1 year (47); the information memory is more accurate. In addition, most of them are supported by physical examination information, and the data on pregnancy, childbirth, and children’s illness is recorded, which can significantly reduce recall bias.

Moreover, some information, such as part of the intrauterine environment and external environmental factors during pregnancy, cannot be collected. Confounding bias cannot be avoided entirely. Therefore, it is urgent to provide more prospective studies with larger samples needed to verify the authenticity of the results and explore the underlying mechanisms.

In conclusion, our study demonstrated that paternal overweight and obesity before pregnancy were associated with an increased risk of maternal-neonatal outcomes with HDCP, cesarean delivery, and macrosomia. More importantly, it may cause poor long-term prognosis in adolescents. It demonstrated that the incidence of dental caries, asthma, and HFMD of adolescents in the paternal obesity group showed an increasing trend. In addition, we also found that the underweight paternal group is a risk factor for the incidence of SGA and anemia in adolescents. All these results demonstrated the adverse effects of paternal overweight and obesity on maternal-neonatal outcomes and long-term prognosis in adolescents. In addition to focusing on maternal weight, expectant fathers should pay more attention to weight management since BMI is a modifiable risk factor. Preventing paternal obesity can lead to better maternal and child outcomes. It would provide new opportunities for chronic diseases.

## Data availability statement

The original contributions presented in the study are included in the article/**Supplementary Material**. Further inquiries can be directed to the corresponding authors.

## Ethics statement

The studies involving human participants were reviewed and approved by The Hospital Ethics Committee of Fujian Maternity and Child Health Hospital. Written informed consent to participate in this study was provided by the participants’ legal guardian/next of kin. Written informed consent was obtained from the individual(s), and minor(s)’ legal guardian/next of kin, for the publication of any potentially identifiable images or data included in this article.

## Author contributions

All authors made a significant contribution to the work reported, whether that is in the conception, study design, execution, acquisition of data, analysis, and interpretation, or all these areas; took part in drafting, revising, or critically reviewing the article; gave final approval of the version to be published; have agreed on the journal to which the report has been submitted; and agree to be accountable for all aspects of the work.
