# Supplementary material for: Effects of paternal obesity on maternal-neonatal outcomes and long-term prognosis in adolescents
Source: Front Endocrinol (Lausanne). 2023 Mar 30;14:1114250. doi: 10.3389/fendo.2023.1114250 (PMC10111374; doi:10.3389/fendo.2023.1114250)
Supplement: Supplementary file 1 [file Table_1.docx]

**Supplementary Table 1: Paternal BMI and the risk of HDCP/Cesarean delivery was analyzed by logistic regression, stratified by maternal BMI.**

| **Maternal BMI** | | **Paternal BMI** | | **Crude** | | **Adjusted** | | *P* for interactionc |
| --- | --- | --- | --- | --- | --- | --- | --- | --- |
|  |  | **Group** | **n(%)** | OR(95%CI)a | ***P* value** | OR(95%CI)b | ***P* value** |  |
| **HDCP** | **Underweight** | Normal | 33 (1.7) | 1(Ref) |  | 1(Ref) |  | 0.214 |
|  |  | Underweight | 3 (2.5) | 1.51 (0.46-4.99) | 0.501 | 1.54 (0.46-5.14) | 0.483 |  |
|  |  | Overweight | 27 (2.1) | 1.24 (0.74-2.08) | 0.403 | 1.29 (0.77-2.16) | 0.337 |  |
|  |  | Obesity | 19 (4.9) | 3.04 (1.71-5.40) | <0.001* | 3.08 (1.71-5.52) | <0.001* |  |
|  | **Normal** | Normal | 183 (1.6) | 1(Ref) |  | 1(Ref) |  |  |
|  |  | Underweight | 14 (2.0) | 1.26 (0.73-2.18) | 0.411 | 1.21 (0.70-2.10) | 0.497 |  |
|  |  | Overweight | 147 (2.1) | 1.31 (1.05-1.63) | 0.016 | 1.27 (1.02-1.59) | 0.034 |  |
|  |  | Obesity | 64 (3.3) | 2.10 (1.57-2.80) | <0.001* | 2.04 (1.53-2.74) | <0.001* |  |
|  | **Overweight** | Normal | 49 (2.1) | 1(Ref) |  | 1(Ref) |  |  |
|  |  | Underweight | 2 (1.3) | 0.59 (0.14-2.46) | 0.472 | 0.61 (0.14-2.53) | 0.493 |  |
|  |  | Overweight | 18 (1.4) | 0.67 (0.39-1.16) | 0.152 | 0.69 (0.40-1.20) | 0.191 |  |
|  |  | Obesity | 9 (2.7) | 1.27 (0.62-2.60) | 0.521 | 1.31 (0.63-2.73) | 0.476 |  |
|  | **Obesity** | Normal | 7 (1.8) | 1(Ref) |  | 1(Ref) |  |  |
|  |  | Underweight | 1 (3.8) | 2.22 (0.26-18.73) | 0.465 | 2.55 (0.26-25.01) | 0.422 |  |
|  |  | Overweight | 3 (1.5) | 0.82 (0.21-3.20) | 0.774 | 0.80 (0.20-3.22) | 0.752 |  |
|  |  | Obesity | 7 (8.4) | 5.11 (1.74-14.98) | 0.003* | 13.29 (3.05-57.93) | 0.001* |  |
| **Cesarean delivery** | **Underweight** | Normal | 651 (33.0) | 1(Ref) |  | 1(Ref) |  | <0.001* |
|  |  | Underweight | 43 (35.8) | 1.13 (0.77-1.67) | 0.522 | 1.11 (0.75-1.64) | 0.607 |  |
|  |  | Overweight | 433 (33.3) | 1.01 (0.87-1.17) | 0.877 | 1.03 (0.88-1.19) | 0.732 |  |
|  |  | Obesity | 116 (30.0) | 0.87 (0.69-1.10) | 0.246 | 0.87 (0.69-1.11) | 0.270 |  |
|  | **Normal** | Normal | 4152 (36.6) | 1(Ref) |  | 1(Ref) |  |  |
|  |  | Underweight | 271 (39.2) | 1.11 (0.95-1.30) | 0.176 | 1.10 (0.94-1.29) | 0.224 |  |
|  |  | Overweight | 2668 (38.1) | 1.07 (1.00-1.14) | 0.036* | 1.06 (1.00-1.13) | 0.066 |  |
|  |  | Obesity | 708 (36.8) | 1.01 (0.91-1.11) | 0.883 | 0.99 (0.90-1.10) | 0.874 |  |
|  | **Overweight** | Normal | 873 (37.7) | 1(Ref) |  | 1(Ref) |  |  |
|  |  | Underweight | 71 (44.9) | 1.35 (0.98-1.87) | 0.070 | 1.38 (0.99-1.91) | 0.058 |  |
|  |  | Overweight | 535 (42.5) | 1.22 (1.06-1.41) | 0.005* | 1.23 (1.07-1.42) | 0.004 |  |
|  |  | Obesity | 190 (56.2) | 2.12 (1.69-2.67) | <0.001* | 2.13 (1.68-2.70) | <0.001* |  |
|  | **Obesity** | Normal | 173 (43.8) | 1(Ref) |  | 1(Ref) |  |  |
|  |  | Underweight | 12 (46.2) | 1.10 (0.50-2.44) | 0.815 | 1.24 (0.54-2.84) | 0.614 |  |
|  |  | Overweight | 77 (37.4) | 0.77 (0.54-1.08) | 0.130 | 0.79 (0.55-1.13) | 0.194 |  |
|  |  | Obesity | 71 (85.5) | 7.59 (3.99-14.45) | <0.001* | 8.67 (4.36-17.22) | <0.001* |  |

BMI, body mass index; HDCP, hypertensive disorder complicating pregnancy; OR, odds ratio;95% CI, 95% confidence interval of the estimated trend.

a Univariate logistic regression compared with the referent (norma paternal BMI).

b Estimated using multivariate logistic regression. Model for HDCP adjusted for Child sex, child age, Paternal age, Paternal education, Maternal age, Maternal education,parity, Gravidity, Annual family income, Cesarean delivery, GWG. Model for Cesarean delivery adjusted for Child sex, child age, Paternal age, Paternal education, Maternal age, Maternal education, parity, Gravidity, Annual family income, HDCP, GWG, gestational age, birth weight, birth height.

c *P* values for Interaction showed the interaction effect of paternal obesity and maternal obesity on the risk of HDCP/Cesarean delivery.

**P* <0.05 was considered statistically significant.
